# A Case of Bronchopleural Fistula and Hydropneumothorax in a Patient With Necrotizing Pneumonia Complicated by Mycobacterium avium Complex

**DOI:** 10.7759/cureus.30280

**Published:** 2022-10-13

**Authors:** Asma U Hosna, Daniel Miller

**Affiliations:** 1 Internal Medicine, Icahn School of Medicine at Mount Sinai, Queens Hospital Center, Jamaica, USA; 2 Internal Medicine, Icahn School of Medicine at Mount Sinai, Queens Hospital Center, Brooklyn, USA

**Keywords:** bronchopulmonary fistula, complications’, supportive treatment, atypical infection, hydropneumothorax, necrotizing pneumonia

## Abstract

A bronchopulmonary fistula is a pathological connection that develops between the bronchi and the pleural cavity as a result of etiologies including surgery, infection, blunt or penetrating trauma, radiation, chemotherapy, and chronic obstructive pulmonary disease sequela. Diagnosis and treatment are challenging for intensivists. We present a case report of bronchopulmonary fistula resulting in hydropneumothorax caused by necrotizing pneumonia and complicated by mycobacterium avium complex that resolved spontaneously. The aim of this case report is to discuss the presentation and treatment of bronchopleural fistulas.

## Introduction

Bronchopulmonary fistula (BPF) has a high morbidity and mortality rate; if left untreated, mortality may exceed 71% [1]. The incidence of BPF is 4.5%-20% after a pneumectomy and 0.5% after a lobectomy [2,3], and infection is also a common cause. The patient described in this study had necrotizing pneumonia complicated by mycobacterium avium complex (MAC) which led to the development of hydropneumothorax. MAC organisms are common in the soil and water and are easily inhaled during daily activities. Usually, they are harmless, but they can cause clinical complications in individuals with certain risk factors, including lung diseases, such as bronchiectasis and chronic obstructive pulmonary disease, and a weakened immune system resulting from an autoimmune disorder or treatment with drugs that suppress immunity. A finding of necrotizing empyema with subsequent culture revealing the presence of MAC organisms makes clear that a patient’s immunocompromised state has contributed to the development of BPF [4]. We present here a case of a bronchopleural fistula resulting in hydropneumothorax caused by necrotizing pneumonia and complicated by MAC. The condition resolved spontaneously after chest tube placement for hydropneumothorax and the mechanism-injury-signs-treatment (MIST) protocol without requiring the placement of an endobronchial valve (EBV).

## Case presentation

**Figure 1:**
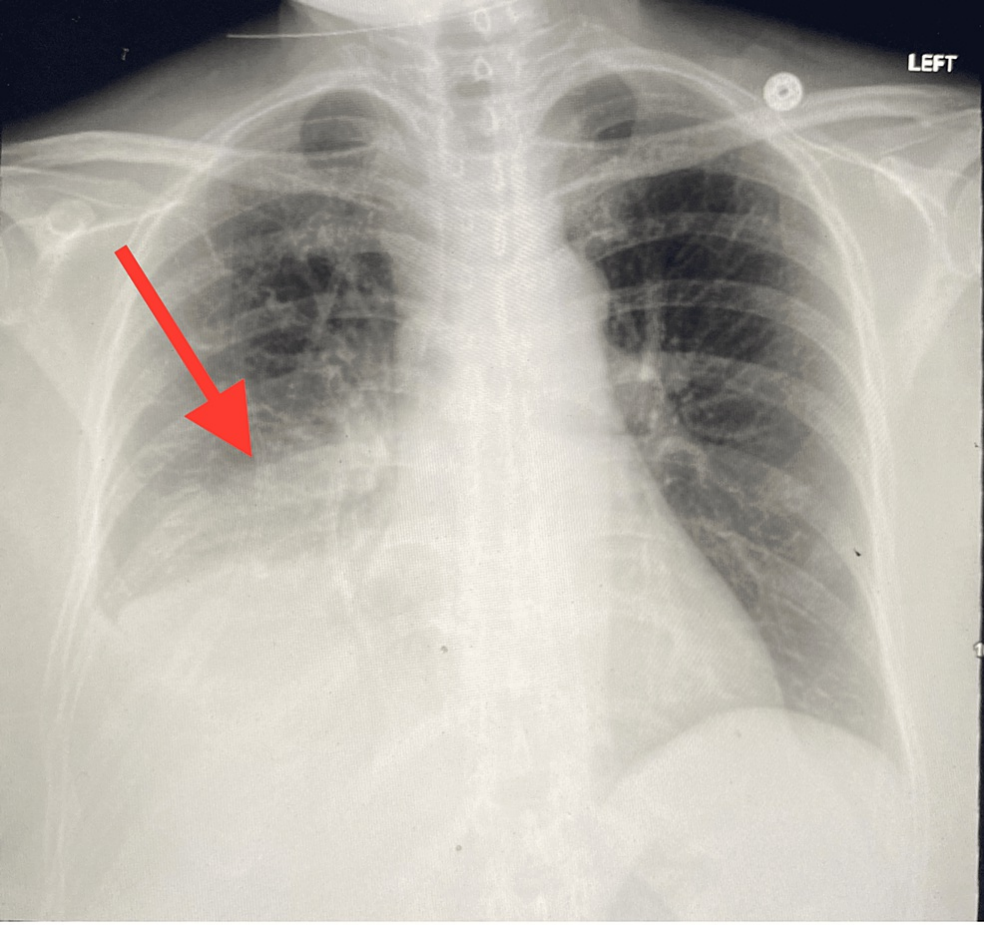
Chest x-ray showing airspace consolidations in the right lower lobe concerning for pneumonia.

**Figure 2:**
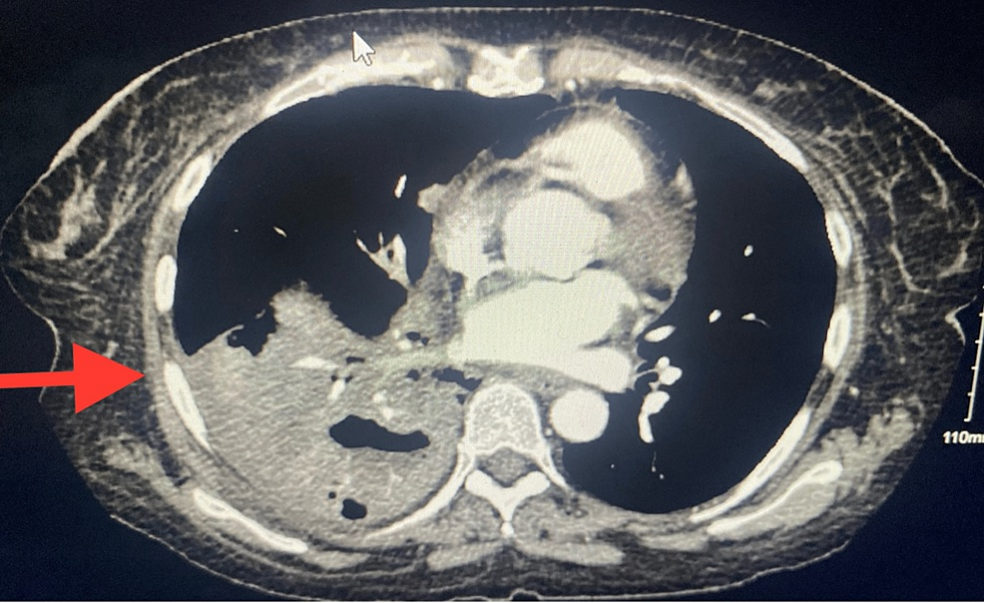
CT chest showing right lower lobe consolidation with cavitation suspect necrotizing pneumonia.

We present a case of a 50-year-old woman with a medical history of asthma (she was intubated once during childhood) and seizure disorder who presented to the ED for worsening cough and shortness of breath. The patient reported chills and cough over the preceding two weeks. The accompanying symptoms included grey-colored sputum that had increased in amount over the previous couple of days and pleuritic chest pain when coughing. She had not monitored her temperature at home but reported feeling feverish. She reports feeling like her health declined since the time she received the COVID-19 vaccine (Pfizer) four months earlier. She believed that the cough had been triggering her asthma symptoms, which she treated with albuterol but to no effect. On examination, the patient was found to be tachycardic, tachypneic, hypoxic to 84% on room air (with an improvement to 98% with 2L O_2_ via nasal cannula), and hypotensive to 96/71 mmHg. On auscultation, the patient had Ronchi on bilateral lower lung bases. The initial laboratory results revealed leukocytosis, anemia, elevated lactate, lymphocytopenia, neutrophilia, hypokalemia, hypophosphatemia, hyponatremia, hypocalcemia, and respiratory alkalosis, and the patient’s procalcitonin, d-dimer, and ferritin levels were elevated. However, her lactate dehydrogenase level was within the normal range (Table 1). Her troponin level was 0.013, and her EKG showed sinus tachycardia with nonspecific T-wave abnormalities. A chest x-ray showed airspace consolidations within the right lower lung field suggesting pneumonia (Figure 1). CTA chest showed right lower lobe consolidation with cavitation suggesting necrotizing pneumonia (Figure 2).

**Table 1:** Lab value reported at the time of patient’s presentation.

Labs obtained at the time of presentation	Lab value	Normal range and reference units
White blood cells	28.13(H)	4.80-10.80 x 10(3)/ mcL
Red blood cells	3.00(L)	4.20-5.40x 10(6)/ mcL
Lymphocytes	4(L)	20.0-45.0%
Neutrophils	89.3(H)	44.0-70.0%
Lactate	2.9(H)	0.6-1.4 mmol/L
Potassium	3.0(L)	3.5-5.1 mmol/L
Phosphate	2.2(L)	2.5-4.5 mg/dL
Sodium	134(L)	136-145 mmol/L
Calcium	8.1(L)	8.6-10.3 mg/dL
Pro calcitonin	0.63(H)	0.02-0.10 ng/mL
D-dimer	730(H)	<250 ng/mL
Ferritin	298(H)	15-150 ng/mL
Ph	7.47(H)	7.32-7.43

**Figure 3:**
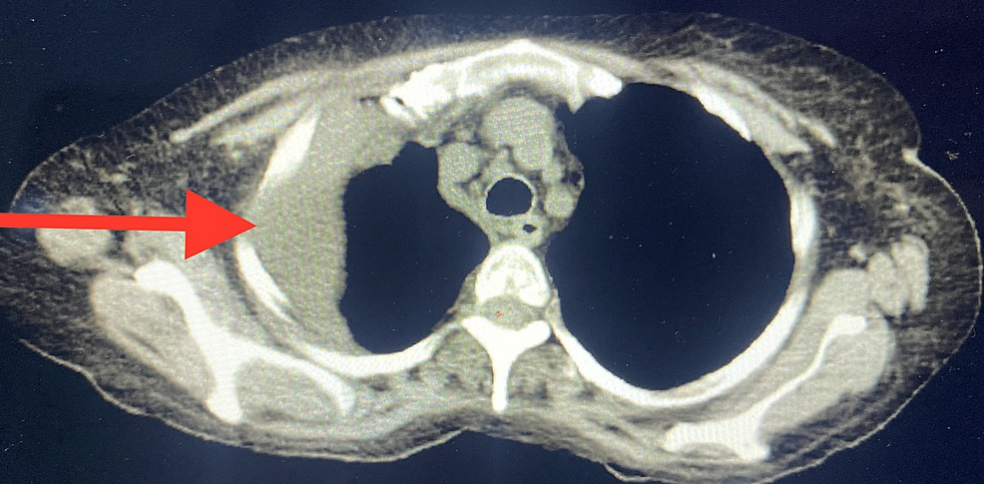
Small hydropneumothorax in which some of the fluid is located. Focal consolidation in right lower lobe may represent atelectasis and/or pneumonia.

The patient was admitted to the medicine floor and started on intravenous piperacillin/tazobactam 3.375 g q 6h hourly, azithromycin 500 mg daily, and vancomycin 1,000 mg q 12th hourly. She also received Normal saline at 100 mL/hour for low blood pressure, albuterol, and Budesonide/formetrol inhaler. Sputum culture showed normal respiratory flora, and an atypical workup for mycoplasma, urine Legionella, and Streptococcus pneumonia was negative. A follow-up CT chest on the fourth day of admission to the hospital showed right-sided hydropneumothorax (Figure 3).

Pulmonary was consulted, a chest tube was inserted and placed on wall suction, and 270 mL of serous fluid was collected. The patient continued to have a productive cough. Repeat sputum culture showed mold-like fungus. Pulmonary reevaluated the patient, and bedside POCUS showed loculated pleural effusion. Repeated CT chest continued to show hydropneumothorax with a small amount of loculated fluid.

**Figure 4:**
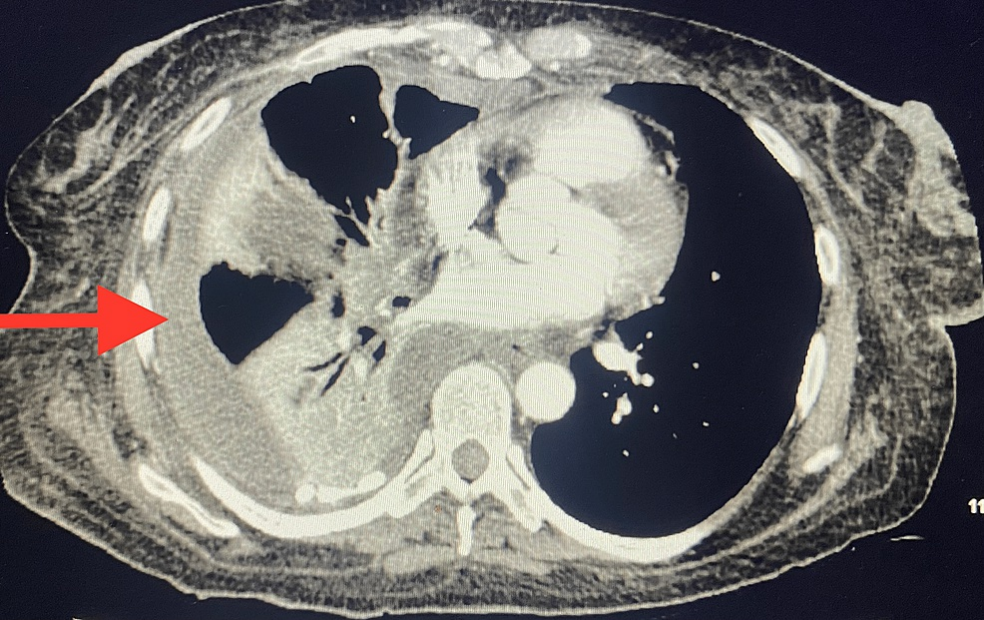
CT chest showing small loculated right-sided pleural effusion, interval development of left-sided pleural effusion, and linear opacities of atelectasis in the right middle lobe. The effusion is slightly smaller than those reported in prior studies. Stable right lower lobe consolidation demonstrates that the heterogeneous enhancement likely contained a small area of cavitation.

In the absence of any obvious bleeding, the patient’s hemoglobin dropped to 6.6, so 1 unit of PRBC was transfused. The Pulmonology team recommended starting the MIST protocol for loculated pleural effusion, the patient was transferred to the step-down unit, and the protocol was started. The patient received the first dose of MIST without complications but, shortly after receiving the second, became hypotensive, tachycardic, and visibly pale. Stat labs were drawn that showed hemoglobin <5 g/dL, so three more units of PRBC were transfused along with one unit of fresh frozen plasma. The hemoglobin level improved after the transfusion, but a large volume of drainage continued from the right chest tube. On the following morning, the patient became hypotensive, with a MAP of 55 mmHg and tachycardia of 130-140 bps. The hemoglobin level dropped below 7 g/dL, and two more units of PRBC and one unit of cryoprecipitate were transfused. The patient was started on peripheral Norepinephrine to support blood pressure. Because of her hemodynamic instability, frequent need for blood transfusion, and bleeding from the chest tube, the patient was upgraded to ICU. An internal jugular line was inserted, and the peripheral Norepinephrine was switched to the central Norepinephrine. The MIST protocol was put on hold because the patient was bleeding from the chest tube. Later, Norepinephrine was discontinued as blood pressure improved, and the patient remained hemodynamically stable. CTA chest revealed a small loculated right-sided pleural effusion and interval development of a left-sided pleural effusion (Figure 4).

Pleural fluid was sent for analysis, which revealed MAC. Accordingly, the patient was started on RIPE therapy. The patient was then downgraded to a step-down unit since her condition was stable. Sputum culture and further pleural fluid analysis were positive for MAC. The patient was encouraged to use a spirometer and undergo daily chest physiotherapy. The output from her chest tube had decreased significantly by this point. A further repeat CT chest showed improvement in pleural effusion.

The patient was downgraded to the medicine floor for further management. Body fluid culture PCR was negative for MAC, and QuantiFERON TB gold was negative, so the RIPE therapy was discontinued. Another repeat CT chest likewise showed significant improvement. The chest tube was then discontinued without complications. Infectious disease was consulted regarding the antibiotic course for necrotizing pneumonia and recommended six weeks of treatment. The antibiotic was switched from vancomycin and Piperacillin-tazobactam and Amoxicillin + clavulanate 500 mg twice daily. The patient was discharged home with outpatient pulmonology follow-up.

## Discussion

A BPF is a connection between the bronchial tree and the pleural space that allows air to leak into the pleural space. BPFs are challenging to manage and are associated with high rates of mortality and morbidity. The condition occurs after 4.5%-20% of pneumectomies and 0.5% of lobectomies [4]. Infection is also a very common cause of BPF.

The complications of BPF include pulmonary space infection, difficulty in lung expansion, and hypoxic respiratory failure. The presentation varies from sudden-onset hypoxia, subcutaneous emphysema, and cough with purulent sputum to persistent air leak, tracheal and mediastinal deviation, shock, and even death [2]. The overall mortality ranges from 25% to 71% [3], with aspiration pneumonia, tension pneumothorax, and ARDS being the most common causes of death Identifying and treating the condition as early as possible is the only means to prevent morbidity and mortality.

Necrotizing pneumonia is a rare complication of bacterial lung infections manifested in multiple cavities within lung parenchyma [5]. Necrotizing pneumonia is commonly associated with an underlying condition and immunocompromised patients. The medical history of the patient described in this case study included asthma and seizures, and she had been intubated during childhood. Her condition rapidly deteriorated in association with the development of hydropneumothorax which later developed into hemopneumothorax, likely as a result of intra-alveolar bleeding.

Contrast-enhanced computed chest tomography is the best diagnostic modality, but bronchoscopy is the gold standard as it helps for both direct visualization of BPF as well as sequential balloon occlusion to access the location in real-time [6]. Other modalities for diagnosing BPF include planar and single-photon emission tomography (SPECT) imaging and computed tomography bronchography (CTB) [2], of which the latter is more useful in visualizing the fistula directly.

The treatment of BPF varies from patient to patient. Adequate pleural drainage placement is the most important step in the management of necrotizing pneumonia. Medical management includes pleural drainage, antibiotic treatment, management of the airway, nutritional support, and fluid management [7]. Surgical management depends on a patient’s condition. There are no specific guidelines for the timing of surgery, but the preference is that it be performed after the patient has become clinically stable so as to provide for a better postoperative outcome. Necrotizing pneumonia, as an infectious process, damages the surrounding tissue, leaving it vulnerable to the formation of cavities and the collection of pus. Therefore, delayed surgery has some benefits in terms of allowing time for perfusion to resolve in temporarily damaged areas and facilitating localization of the area requiring permanent resection [8]. Persistent BPF requires EBV placement. Delayed treatment can result in contralateral lung contamination, multiorgan failure, severe sepsis, and even death.

In the patient described here, the appropriate antimicrobial coverage was provided, a chest tube was inserted, and pleural drainage was allowed until her symptoms resolved, so no surgical treatment was required. A repeat CT scan after the removal of the chest tube showed significant improvement in her condition. This case report makes clear that physicians must be aware of severe and complicated pneumonia that unusual pathogens such as MAC can cause and may not respond to conventional treatment. Surgery is not required for every patient but, when it is, timing is the most important factor in determining whether postoperative complications will develop.

## Conclusions

BPF is a common complication of necrotizing pneumonia. Management of it is highly case-dependent, being based on the pathophysiology and severity of the condition. The timing of diagnosis-associated conditions and the severity of the presentation determine the effectiveness of treatment. Therefore, early diagnosis and prompt treatment are the keys to success.
